# Implementation of Video Visits During COVID-19: Lessons Learned From a Primary Care Practice in New York City

**DOI:** 10.3389/fpubh.2020.00514

**Published:** 2020-09-17

**Authors:** Sanjai Sinha, Lisa M. Kern, Laura F. Gingras, Evgeniya Reshetnyak, Judy Tung, Fred Pelzman, Thomas A. McGrath, Madeline R. Sterling

**Affiliations:** ^1^Division of General Internal Medicine, Department of Medicine, Weill Cornell Medicine, New York, NY, United States; ^2^Department of Medicine, Weill Cornell Medicine, New York, NY, United States

**Keywords:** telemedicine, video visit, ambulatory care, implementation science, primary care, COVID-19

## Abstract

**Background:** During the height of the coronavirus (COVID-19) pandemic, there was an unprecedented demand for “virtual visits,” or ambulatory visits conducted *via* video interface, in order to decrease the risk of transmission.

**Objective:** To describe the implementation and evaluation of a video visit program at a large, academic primary care practice in New York, NY, the epicenter of the COVID-19 pandemic.

**Design and participants:** We included consecutive adults (age > 18) scheduled for video visits from March 16, 2020 to April 17, 2020 for COVID-19 and non-COVID-19 related complaints.

**Intervention:** New processes were established to prepare the practice and patients for video visits. Video visits were conducted by attendings, residents, and nurse practitioners.

**Main measures:** Guided by the RE-AIM Framework, we evaluated the Reach, Effectiveness, Adoption, and Implementation of video visits.

**Key results:** In the 4 weeks prior to the study period, 12 video visits were completed. During the 5-weeks study period, we completed a total of 1,030 video visits for 817 unique patients. Of the video visits completed, 42% were for COVID-19 related symptoms, and the remainder were for other acute or chronic conditions. Video visits were completed more often among younger adults, women, and those with commercial insurance, compared to those who completed in-person visits pre-COVID (all *p* < 0.0001). Patients who completed video visits reported high satisfaction (mean 4.6 on a 5-point scale [*SD*: 0.97]); 13.3% reported technical challenges during video visits.

**Conclusions:** Video visits are feasible for the delivery of primary care for patients during the COVID-19 pandemic.

## Background

Shortly after its first confirmed case on March 1, 2020, New York City became the epicenter of the novel coronavirus, SARS-CoV-2, pandemic in the United States (1). As social distancing became a key public health strategy to minimize viral transmission, medical centers, and physician practices were urged to rapidly implement new models of healthcare delivery which met patients' needs, but also limited exposure risk (2). As a result, there was a demand for virtual care, especially video visits, as an alternative to traditional in-person care (3).

Although video visits have been previously used and have been found to be feasible (4), their adoption and utilization have been limited (5–7). As of 2019, only 8% of Americans had ever done a video visit with a physician (8). Reasons for low adoption rates had included: lack of reimbursement, inadequate digital infrastructure, and incompatible workflow (6, 9). For patients, language barriers and inadequate access to technology platforms and the internet (10, 11) were often cited as barriers to video visits (6).

Nevertheless, given the need to care for patients remotely during COVID-19, the demand for video visits increased, potentially outweighing many of these prior utilization barriers. Herein we report the experiences of one large, academic, urban primary care practice with implementing a video visit program during the COVID-19 pandemic in New York City. Using a modified version of the RE-AIM Framework (12), we report on the Reach, Effectiveness, Adoption, and Implementation of video visits in order to describe our experiences to other primary care practices across the country who may need to adopt a similar care model.

## Methods

### Setting and Patient Population

This is a retrospective case study of consecutive adults scheduled for video visits during the 5-weeks period from Monday, March 16, 2020 to Friday, April 17, 2020 at Weill Cornell Internal Medicine Associates (WCIMA). WCIMA is a large, academic, hospital-based primary care practice of Weill Cornell Medicine and NewYork-Presbyterian Hospital (https://weillcornell.org/wcima). As a high-volume tertiary-care clinic, it averages 53,000 office visits per year and serves a diverse patient population (13). At WCIMA, 31 attending physicians, 11 nurse practitioners, and six registered nurses provide care, alongside 129 residents and interns. Because we report on the video visit technology itself, as well as the effectiveness of its implementation, this study is a Hybrid Type 2- Effectiveness Implementation design (14, 15).

### Context: Limited Use of Video Visits Pre-COVID-19

Video visits were first introduced at WCIMA in September 2019. However, only a few providers utilized this technology with their patients. Barriers for use pre-COVID-19 included: limited understanding and training on the technology among providers and staff and uncertainty about how best to divide clinician time between video and in-person visits. To address these barriers, training sessions demonstrating how to schedule and perform video visits occurred during faculty meetings in the fall of 2019 and providers were asked to complete training modules. Despite these efforts, adoption remained low.

### Developing Infrastructure for Video Visits During COVID-19

WCIMA utilized the Epic system for video visits [Epic Systems, Verona, WI]. Patients connected to video visits through a Weill Cornell Connect App on their smartphone (any brand) or tablet whereas providers conducted video visits through Epic Haiku (iPhone) or Epic Canto (iPad). The practice purchased iPads for providers without an iPhone or iPad to use. During or after visits, providers documented encounters using traditional Epic notes via their phone, tablet, or desktop.

**Figure 1:**
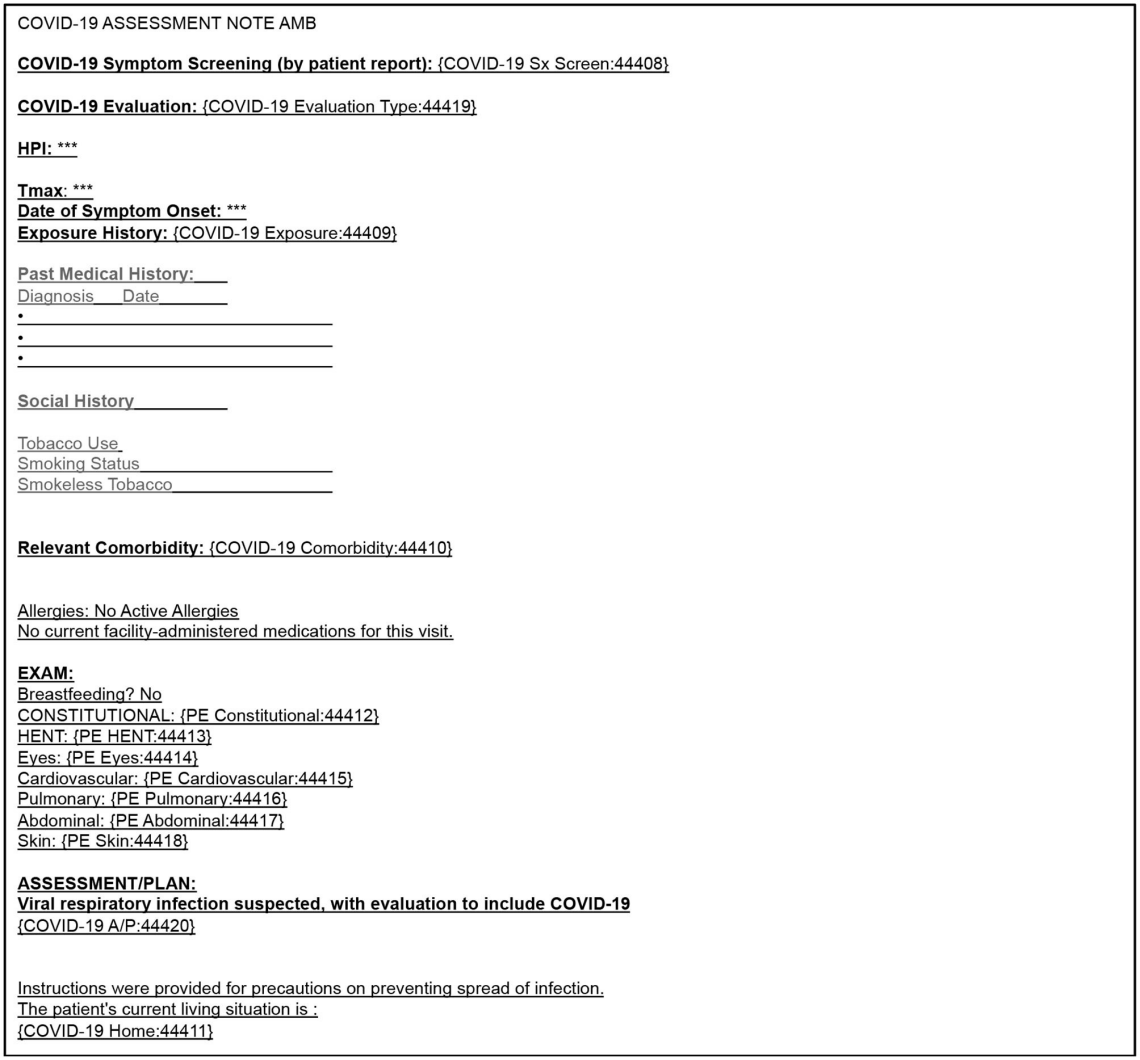
COVID-19 assessment ambulatory note template to accompany video visit encounters.

To minimize COVID-19 transmission and the use of personal protective equipment, starting on March 16, 2020, the majority of care at WCIMA was transitioned to video visits. Providers conducted video visits for patients with COVID-19-like symptoms, as well as for patients with acute and chronic care needs. While providers were encouraged to maximize the use of video visits, they were permitted to conduct in-person visits for urgent complaints where they deemed a physical exam was needed. To guide providers on how to conduct video visits for COVID-19 and usual care, a group of physicians developed a Video Visit Handbook (Appendix 1). This handbook was updated twice during the study period to reflect rapidly changing clinical recommendations for the ambulatory management of COVID-19. Weill Cornell's Physician Organization Information Services (POIS) created an electronic health record template for COVID-19 assessments (Figure 1). Providers were asked to use this template for all COVID-19 related video and in-person visits. As shown in Figure 1, the template contained 10 structured data elements (free-text or drop-down) for the COVID-19 video visit. In addition, POIS developed smartphrases in Epic for patient instructions, based on CDC guidelines for COVID-19 including how to socially distance, and perform self-care monitoring. These smartphrases were developed in English and Spanish.

### Video Visit Workflow During COVID-19

To prepare for the shift to video visits, starting on March 16, 2020, clinic staff received training on how to schedule patients for video visits, including how to teach patients to download and use the app. Each week during the study period, staff and providers reviewed upcoming scheduled visits for that week, and determined who should be seen in person and who could be converted to a video visit. Those eligible for a virtual visit but without access to a smart device and internet connection were offered a telephone visit. A hybrid scheduling model was used, in which providers had half-day sessions devoted to seeing their own patients virtually and others for which they were available for video visits with any patient in the practice, to maximize access. Similar to in-person visits, video visits were 20 min in duration, but could be longer at the discretion of the provider. Video visits took place Monday through Friday, with occasional Saturday visits. Providers conducted video visits from WCIMA offices or remotely. Practice administrators worked with hospital compliance to understand billing procedures, and physicians were trained to document and bill for video visits in accordance with the new rules regarding broadened telehealth payment policies during COVID-19 from the Centers for Medicare and Medicaid Services (CMS) (16). Documentation of verbal consent from the patient to engage in a telemedicine visit was required in each note. The same evaluation and management codes and the same rules for determining level of service for in-person care were used for video visits.

As outlined in the Video Visit Handbook, a main goal of each video visit for COVID-19 was for providers to determine if patients could be managed safely at home with supportive care, if they needed to be evaluated in-person at WCIMA's newly established cough, cold, or fever clinic, or if they needed to go to the emergency room. Another goal was to provide counseling on management of symptoms, warning signs of clinical deterioration, and prevention of transmission. The goal of non-COVID-19 video visits was to approximate traditional, in-person care.

Although physical examinations were limited by the video visit format, providers were able to assess a patient's general appearance, respiratory effort, and affect. When indicated, providers could visually examine patients' skin, sclera/conjunctiva, and the oropharynx. A limited neurological examination could also be performed. Heart rate and respiratory rate could be measured by the patient with provider guidance. For patients with home blood pressure monitors or pulse oximeters, additional vital signs could be collected.

Depending on the visit type, providers chose to document the visit using the COVID-19 assessment template or the usual primary care assessment template.

### Quantitative Data Collection

We used a modified version of the RE-AIM Framework to describe the implementation of video visits at WCIMA during the COVID-19 pandemic (12). As such, we evaluated the Reach, Effectiveness, Adoption, and Implementation of the video visit initiative. We plan to collect data on Maintenance in the future. De-identified, practice-level data were generated from our electronic medical record and billing data.

To assess Reach, we obtained data on the number of completed video visits over time during the study period, demographics (age, gender, race, ethnicity, insurance type, relationship to practice) of the patients seen via video, the level of service of these video visits, and the most frequent diagnoses for which these video visits were billed.

To assess Effectiveness, we collected data on the proportion of scheduled visits that were: completed (as above), failed (due to technical difficulties), canceled, or no-shows. During the study period, but separate from our work, the Weill Cornell Physician Organization conducted a satisfaction survey via email among patients who completed video visits within the Department of Medicine. From this survey we obtained aggregate responses from patients for video visits conducted by WCIMA providers. The survey asked patients to: (1) rate their video visit experience (5-point Likert scale; one worst, five best); (2) report if they were satisfied with the care they received, compared with in-person visits (yes/no); (3) report what percent of care they would like to have as video visits in the future, compared to in-person (fill in %); and, (4) report if technical challenges occurred during the video visit (yes/no).

To assess Adoption, we collected data on the number of staff who assisted with video visit scheduling and the number and types of providers conducting video visits (attending vs. resident vs. nurse practitioners).

To assess Implementation, we collected data on the frequency with which the COVID-19 template was used and the number of iPads our clinic purchased to conduct video visits.

Because this study used de-identified, practice-level data, it was deemed exempt by the Weill Cornell Medicine Institutional Review Board. As such, written informed consent was not required for participation in this study.

### Data Analysis

We present absolute counts and percent frequency of occurrence. We used the Kruskal-Wallis test to evaluate differences between median age categories, and the chi-square test to compare proportions. Analyses were conducted using the software package R (version 3.4.1, Vienna, Austria).

## Results

**Figure 2:**
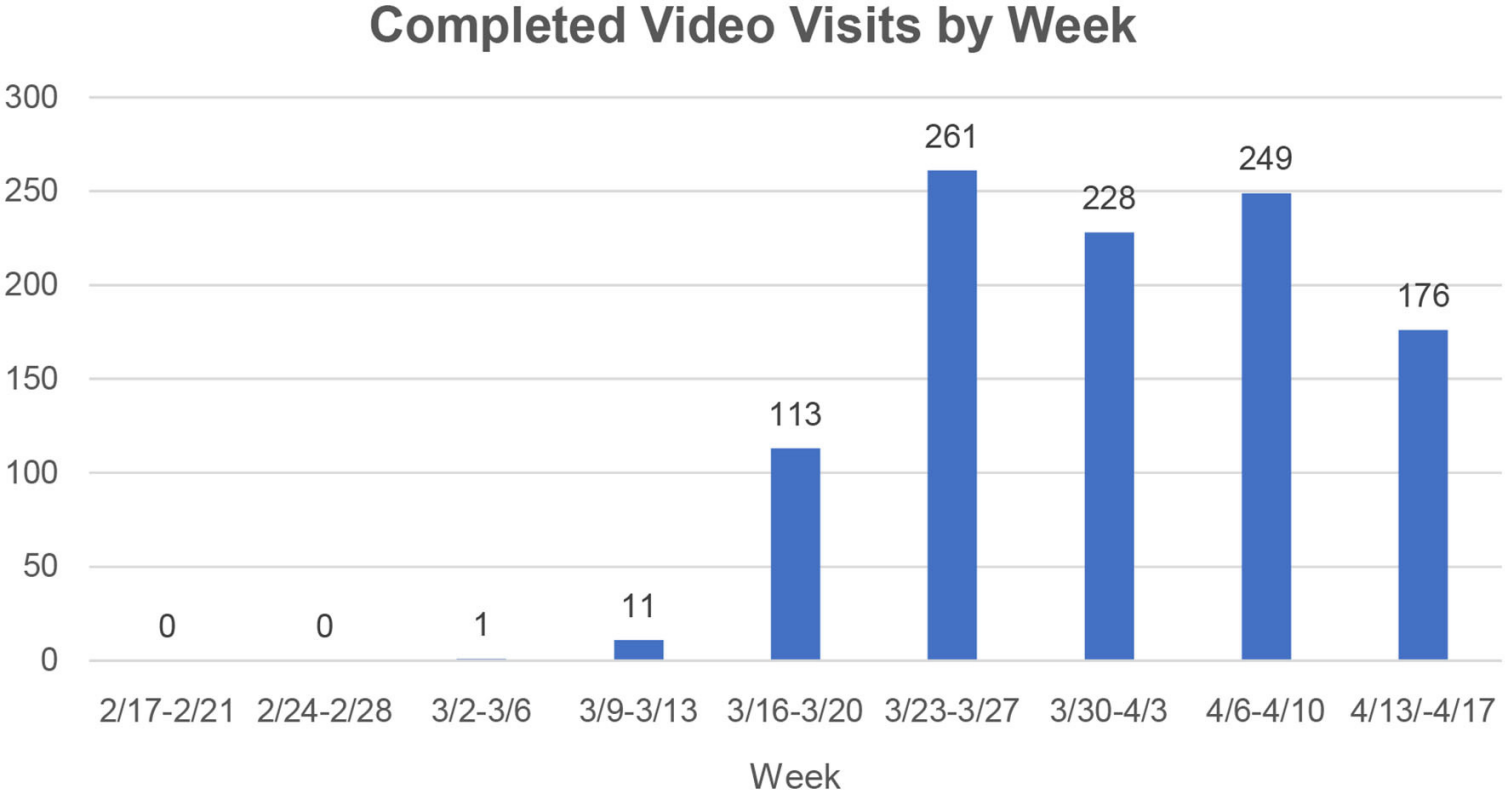
Number of completed video visits by week pre-COVID-19 and during COVID-19. Video Visit Program began during week of 3/16-3/20 in response to the COVID-19 pandemic.

In the 4 weeks prior to the study period, 12 video visits were completed at WCIMA, with 6 (50%) occurring 1 day before our study period. During the 5-weeks study period, we completed a total of 1,030 video visits. The number of these visits by week is shown in Figure 2. In week 1, 113 video visits were completed, followed by 261 in week 2, 228 in week 3, 249 in week 4, and 176 in week 5.

### Reach

Of the 1,030 completed video visits, 817 unique patients participated. Of these, 675 patients (82.6%) had 1 video visit each whereas 142 (17.4%) had >1 video visit (range: 2–9.) The demographics of patients with completed video visits are shown in Table 1. They had a median age of 50 years (Interquartile range: 40.6–61.3), 69% were women, nearly 25% were African American, 23% were Hispanic, 49% had commercial insurance, 28% had Medicaid, and 13% had Medicare. Compared to patients who completed in-person visits in our practice during July 1, 2019 to February 29, 2020 (our fiscal year-to-date data prior to the study period), those who completed video visits were younger (median age of 41–50 vs. 61–70 years [*p* < 0.0001]). The video visit group included more women (69 vs. 65%, *p* = 0.004), more Non-Hispanics (61 vs. 51%, *p* < 0.0001), more Whites (39 vs. 35%, *p* = 0.008), more commercially insured patients (49 vs. 36%, *p* < 0.0001) and fewer Medicare patients (13 vs. 32%, *p* < 0.0001) than our baseline population. Approximately one-fourth (28%) of the video visit group were insured by Medicaid, similar to our baseline population, of which 31% are insured by Medicaid (*p* = 0.158).

**Table 1:** Demographic characteristics of patients who completed video visits during the study period compared to patients who completed in-person visits during the prior fiscal year.

**Characteristics**	**Completed video visits during study period (*n* = 1,030)**	**Completed in-person visits prior to study period (*N* = 38,614)**	Omnibus *p*-values^*^	**Bonferroni adjusted *p*-values for multiple comparisons**
Age, median category, y.	41–50^**^	61–70	<0.0001	
**Sex**				
Male	30.8%	35.2%	0.0035	
Female	69.2%	64.8%		
**Race**				
Black	24.7%	22.0%	0.00003	0.156
Asian / Pacific Isl.	7.9%	8.0%		0.990
White	39.1%	34.5%		0.008
Other/unknown	28.1%	35.2%		<0.00001
**Ethnicity**				
Hispanic	22.9%	19.8%	<0.00001	0.042
Non-Hispanic	61.0%	50.5%		<0.00001
Other/unknown	16.1%	29.7%		<0.00001
**Insurance**				
Medicare	13.0%	31.5%	<0.00001	<0.00001
Medicaid	27.6%	30.6%		0.158
Commercial	49.0%	36.5%		<0.00001
Self-pay/other	10.4%	1.4%		<0.00001
**Relationship to Practice**				
New patient	2.3%	9.3%	<0.00001	
Established patient	97.7%	90.7%		

* *Two-tailed chi-square test*.

** *Median age for adults who completed video visit was 50.0 years (Interquartile range: 40.6–61.3)*.

### Effectiveness

During the study period, 1,475 video visits were scheduled, of which 1,030 (69.8%) were completed and 30 (2.0%) failed due to technical problems and were converted to telephone visits. A total of 19.1% of scheduled video visits no-showed and 9.1% were canceled either by the patient or provider.

Satisfaction data was obtained for 113 (13.8%) of the 817 patients who completed 1 video visit. Patients reported high satisfaction with their video visit (mean score of 4.6 on a 5-point scale [*SD*: 0.97]) and the vast majority (94.5%) of patients were satisfied with the level of care they received during their video visit compared with prior in-person visits (Table 2). Overall, patients preferred that 49% (*SD* 0.26) of future encounters with their provider be video visits instead of in-person visits. A total of 13.3% reported technical challenges during the video visit.

**Table 2:** Patient satisfaction and attitudes toward video visits.

**Survey domain**	Response (*n* = 113)^*^ (mean [*SD*]) or %
Overall experience with video visit (one worst, five best)	4.6 (0.97)
Satisfied with the level of care offered during video visit, compared with in-person visit?	94.5%
Percent of future care preferred as video visit vs. in-person	49% (0.26)
Experienced technical challenges during video visit	13.3%

* *13.8% survey response rate*.

### Adoption and Implementation

Overall, 70 providers (23 attendings, 38 residents, and nine nurse practitioners) conducted these 1,030 video visits and 22 staff members helped orient and schedule patients to video visits (Table 3). Among the video visits completed, the majority (92%) were associated with level 3 and four billing codes, indicating moderate complexity. Review of ICD-10 codes associated with primary billing diagnoses revealed that 428 encounters (42%) were potentially covid-19 related with diagnoses including: cough, upper respiratory infection, fever, chills, shortness of breath, anosmia, wheezing, pneumonia, asthma, musculoskeletal pain, and COVID-19. Overall, 22.9% of video visits used the COVID-19 template. A total of 17 iPads were purchased during the study period.

**Table 3:** Description of providers, level of service, and template usage for completed video visits.

**Characteristics**	**Video visits during COVID (*n* = 1,030)**
**Provider rendering video visit**	
Attending (MD)	69.1%
Nurse Practitioner	8.5%
Resident	22.3%
Level of service of visit^*^	
99202/99212	1.9%
99203/99213	44.5%
99204/99214	47.7%
99205/99215	2.3%
Other^**^	3.4%
COVID-19 structured template used	22.9%

* *Level of service of visit: These CPT codes for ambulatory visits denote whether the patient is new or established and the complexity of medical decision making*.

** *Other includes visits for preventative health, smoking cessation, anticoagulation counseling and psychiatric illness. “Failed” video visits (N = 30), which were converted to telephone only, were not counted in denominator*.

## Discussion

This study describes the implementation of a video visit program at a large academic hospital-based primary care practice in New York City during the COVID-19 pandemic. Overall, 70 providers completed a total of 1,030 video visits over a 5-weeks period, compared to 12 video visits completed in the preceding 4 weeks. Video visits increased greatly during weeks 1 and 2, plateaued in weeks 3 and 4, and dropped off in week 5, which may reflect the overall trend of health care utilization for COVID-19 in New York City during this time (17). Video visits occur more often among younger adults, women, and those with commercial insurance, compared to those with in-person visits pre-COVID. Although we were only able to obtain satisfaction data on a subset of patients, the majority reported high satisfaction with their video visit experience.

Another key finding was that despite our quick ramp-up period, issues with the technology itself among those with scheduled visits appeared to be modest. For example, only 2% of initiated video visits were not completed and converted to telephone encounters. Additionally, only 13% of patients reported challenges with technology during their video visit encounter. Although we could not assess the number of patients who did not engage with video visits, our findings suggest that video visits may be more feasible than previously thought. That is, prior studies have found patients were uncomfortable with the technology and had technical issues during video visits (18, 19). We hypothesize that during COVID-19, patients and providers may have been more willing to engage with and troubleshoot technological challenges in order to be seen. Of note, during the study period, our video visit no-show rate was 19%, which is similar to our in-person no-show rate of 20%. All told, these data signal that in the context of social distancing, and with appropriate workflow and administrative processes, implementation of video visit technology is feasible in primary care.

To our knowledge, this is one of the first studies to describe the implementation of video visits during COVID-19 in the primary care setting. There have been studies describing the implementation of video visits pre-COVID-19 across a range of clinical specialties (20) as well as three studies describing the implementation of video visits during COVID-19, one among an inpatient urology consultation service (21), one among an obstetrics practice (22), and another in urgent care (23). A recent NEJM Catalyst article (3) qualitatively described the experiences of four primary care practices who have gone “virtual” since COVID-19. Like us, they report low utilization of video visits pre-COVID and high utilization during COVID-19. Our study adds to this body of literature by offering a detailed description of video visit implementation, including a Video Visit Handbook, as well as data on reach, effectiveness, and adoption.

Our findings not only have implications for clinical care and healthcare delivery during COVID-19, but also raise questions about the utilization of video visits in primary care moving forward. First, although we lack data on our entire sample, satisfaction scores for video visits were high and patients preferred to have half of their future visits occur via video, compared to in-person. Future research will need to determine if this preference persists after social distancing policies are relaxed. Additionally, studies are needed to assess providers' perceptions regarding the clinical effectiveness of video visits for COVID and non-COVID symptoms. Second, although video visits minimized transmission risk, they also limited the ability of providers to perform a complete physical examination and measure vital signs. Some practices, including ours, have incorporated aspects of remote monitoring into video visit encounters (24). Moving forward, key questions include: *How to best deploy this equipment to patients? Which patients benefit from monitoring? How can these data be captured electronically? Will the cost of such devices will be reimbursable* (25, 26). Third, attention to who is not utilizing video visits will be important to avoid exacerbating existing inequities in health and healthcare. We found that older adults and Medicare beneficiaries were less likely to engage, which may be due to difficulties with technology, lower levels of internet use, sensory impairments, or lack of confidence with technology (10, 11, 27). It is also likely those with limited English proficiency and health literacy, as well as structurally disadvantaged populations, may lack the ability and/or resources to access technology (28). Understanding these barriers will be critical for more equitable implementation. Fourth, primary care physicians may need to create new processes and pathways with specialty providers to co-manage COVID-19 patients who have persistent or multi-organ complications (29, 30). Finally, as CMS and other payers broaden their telehealth payment policies during COVID-19, the economic impact of expanding video visit use will need to be monitored.

### Limitations

Patient satisfaction data was only available from some patients who utilized video visits, which may introduce response bias. Additionally, we do not yet have outcome data on patients with completed video visits. Finally, this study did not include the perspectives of providers and staff on implementing video visits.

## Conclusion

At the height of the COVID-19 pandemic in New York City, we implemented a video visit program at our primary care practice to evaluate and treat patients for their symptoms while maintaining social distance. During a 5-weeks period, 70 providers completed 1,030 video visits, compared to only 12 video visits completed in the preceding 4 weeks. New workflows for staff, providers, and patients were developed to implement this program. Overall, patients reported high satisfaction with the care they received during their video visits. Our findings suggest that video visits provide a feasible way to care for patients with and without COVID-19 symptoms. Additional study on the sustained implementation of video visits in primary care, as well as their effect on patient outcomes, is warranted.

## Data Availability Statement

The raw data supporting the conclusions of this article will be made available by the authors, without undue reservation.

## Ethics Statement

Ethical review and approval of our study was granted through the Weill Cornell Institutional Review Board. Because our study used de-identified, practice-level data, it was deemed exempt from full board review based on federal regulations. As such, written informed consent was not required for participation in this study.

## Author Contributions

SS, LK, LG, and MS were involved in the study design and methods. ER, MS, and SS were involved in the data analysis. All authors contributed sufficiently to the writing and editing of this article.

## Conflict of Interest

LK is a consultant to Mathematica, Inc. SS is a consultant for EverydayHealth, Inc. and Sharecare, Inc. The remaining authors declare that the research was conducted in the absence of any commercial or financial relationships that could be construed as a potential conflict of interest.
