# Supplementary material for: Implementation of Video Visits During COVID-19: Lessons Learned From a Primary Care Practice in New York City
Source: Front Public Health. 2020 Sep 17;8:514. doi: 10.3389/fpubh.2020.00514 (PMC7527590; doi:10.3389/fpubh.2020.00514)
Supplement: Supplementary file 1 [file Data_Sheet_1.pdf]

Video Visit Handbook for WCIMA Providers

Video visits: Video visits are medical encounters that are conducted via video software, rather than in-person. At Weill Cornell Medicine, patients connect through their Weill Cornell Connect App on their smartphone (any brand) or tablet. The WCIMA staff will provide patients directions for how to connect (*you* as a provider do not have to do this). Providers conduct the video visits through Epic Haiku (iPhone) or Epic Canto (iPad). To conduct a video visit as a provider, you will need to use one of these devices/platforms (which must be tagged by ITS at WCM) or through one of several iPads located at WCIMA. An Android device will *not* work for a provider, even if tagged. Before conducting a video visit, you must complete the Epic training module for clinicians. When doing a video visit, please consult the COVID-19 Ambulatory Testing and Management Protocol (last revised April 10, 2020), available on the NYP Infonet.

Types of video visits: Currently at WCIMA, a provider scheduled to perform video visits is likely to encounter patients scheduled for several reasons. ***This document walks you through the anticipated management and workflow for each of these visits*.**

Video Visit Type # 1 – Chronic disease visit with a WCIMA patient

This video visit is for a WCIMA patient who needs to be seen for management of one or more of their chronic conditions (Ex: 50 y.o man with HTN, OA, and IDDM, here for diabetes management)

- History and exam: Conduct a thorough history, as you would in the office. For vitals and the exam, do what you can and be creative. Ex: watch them breathe for respirations; ask them about their home BPs if they have blood pressure cuffs; comment on general appearance, etc. Unfortunately, you will need to enter a pain score for the encounter.
- Labs - Most lab work can be deferred (remember, we are trying *to limit* the number of people we are bringing into clinic); in this example, ask a patient with diabetes to review their blood sugar log with you during the video visit and adjust insulin dosing accordingly. *If you need to send labs*, you can a) order them as you usually would, but order them as future labs and route a message to the WCIMA nursing pool ("*p wcima nurses*") to notify the nurses that the patient was instructed to come in. The nurses can then reach out to the patient to instruct them optimal times to come in (goal is to limit the number of patients in clinic at any point in time) or b) for patients in the outer boroughs who cannot come into WCIMA safely, we now have a bidirectional interface with LabCorp and Quest. Order as future lab collect (select either LabCorp or Quest) and patients can go to their local station and have their blood drawn. The results will file back into Epic.
- Medications – prescribe more liberally than usual (Ex: refill Synthroid without the usual TSH in the last 12 months), but do not refill certain high-risk medications that are usually only prescribed by specialists (Ex: immunosuppressants). You will need to do a medication reconciliation for the encounter as you normally would.
- Documentation: Write a complete note with HPI, ROS, Vitals/Exam, and assessment and plan (as you normally would). Note that for a video visit, you must include a statement at the top of the visit that the patient consented to video visit.

Video Visit Type # 2 – Sick visit with a WCIMA patient (non-COVID)

This video visit is for a WCIMA patient who needs to be seen for an acute complaint, which may or may not be related to their chronic conditions (Ex: 70 y.o. man with non-valvular AFib on coumadin hits his head on a shelf unloading groceries)

- History and exam: Take a focused history and conduct as much of a relevant physical exam as possible (Ex: a neuro exam is indicated - do what you can through the video screen).

- Labs – see visit # 1
- Imaging – If you feel the patient requires imaging, order it as usual (in this case, "Head CT w/o IV contrast") but ask the patient to call radiology 212-746-6000, as they will need an appointment. NOTE: Radiology is no longer taking walk-ins even for Xrays.
- Medications – see visit # 1
- Documentation – see visit # 1

Video Visit Type # 3 – Sick visit with a WCIMA patient for COVID-19-like symptoms

This video visit is for a WCIMA patient who needs to be seen for COVID-19 like symptoms. The goal of the video visit is to triage the patient to the right setting. Patients require significant amount of education, reassurance and concrete home isolation advice.

- Before starting the visit: Review the COVID-19 Ambulatory Testing and Management Protocol and have it near-by (last updated April 10, 2020). This will help focus your questions during the visit.
- History: Conduct a focused history and do as much of the exam as possible (Ex: *does she look sick vs. not sick? Is she struggling to breathe or talking in full sentences comfortably?*). Look through the chart and ask them about their medical history/chronic conditions (this is important for management). Ask about exposures to confirmed or suspected COVID-19 patients. Inquire about their living situation. Use the **COVID Ambulatory Assessment smart text** in Epic to guide your questions and to allow for retrospective collection of information about our COVID related visits.
- Deciding how to manage the patient: *You will need to decide whether this person falls into one of three categories*: ***(a)*** Stay home and isolate, with follow-up as needed;
- ***(b)*** Come into WCIMA for a face-to-face in Cough/Cold/Fever (CCF) clinic (where COVID-19 testing can be done as well as XRAY and labs) (see below); or
- ***(c)*** Send to the emergency department (ED). *Remember, we do not have enough tests for everyone and the majority of patients can be safely managed at home. Bringing them in may unnecessarily expose others.* If the patient will stay home, ask them to call back if symptoms worsen or add them to our **follow-up list (**see below) for closer monitoring.
- Medications: You may empirically prescribe treatment. Examples: Tylenol, Tamiflu [if you suspect influenza or co-occurring influenza]), or antibiotics if you suspect a bacterial infection. You will need to do a medication reconciliation (click 'reviewed meds') for the encounter. Advise Tylenol over NSAIDS for fever management.
- Counseling for isolation with and without testing: There are counseling guidelines available in Epic (English and Spanish) located in the 'Patient Instruction' section. (COVID Instructions- Not Tested). Although it may seem early, try to discuss goals of care with patients so we can document this now, should they do poorly later.
- Patient letters for employers: Use the following template: *.WCIMACOVIDSYMP*
- Face-to-Face Visit at WCIMA CCF clinic: If you feel the patient needs to be seen at WCIMA, *route a message to* ***"p wcima blue front desk*****"** indicating this need. The staff will then call the patient with an appointment date/time and place the patient on the CCF schedule which is labelled 'WCIMA Overflow' schedule. Label the reason for call in the telephone encounter as *"covid-19 evaluation."*
- Documentation and orders: Use the *COVID ambulatory assessment smart text* and smart set in Epic for COVID. These will provide a structured note template as well as the diagnosis codes and patient instructions on how to manage their symptoms at home.
- Follow-up: For patients you want followed by nurses and medical students (with **two follow-up calls at 48 hours and 5 days**) add the patient *to "PATIENT LISTS-> WCIMA COVID Nursing Initial->"*

Video Visit Type # 4 – Patient with COVID-19-like symptoms referred to WCIMA from one of the *non-medicine* specialty practices

This video visit is for a *non-WCIMA patient* who needs to be seen for COVID-19 like symptoms. This patient has been previously seen at Weill Cornell by a non-medicine specialty practice.
History: Identical to Video Visit Type # 3, BUT, they are new to WCIMA and documentation on their chronic conditions and other pertinent information may be absent from the chart. Therefore, try to gather this and document it.

- Deciding how to manage the patient: See Video Visit # 3; follow same algorithm.
- Medications: See Video Visit # 3
- Counseling for isolation with and without testing: See Video Visit # 3
- Face-to-Face Visit at WCIMA Fever/cough clinic: See Video Visit # 3; staff will tell patient where we are located.
- Documentation: – see visit # 3.
- Follow up: - see visit # 3

Video Visit Type # 5 – Patient with COVID-19-like symptoms referred to WCIMA from one of the *medicine* sub-specialty practices

This video visit is for a *non-WCIMA patient* who needs to be seen for COVID-19 like symptoms. This patient has been previously seen at Weill Cornell by a medicine sub-specialty group. Their triage to WCIMA *may* reflect a need for an in-office evaluation, as compared to patients referred from non-medicine specialists.

- History taking: Identical to Video Visit Type # 3, but they are new to WCIMA and documentation on chronic conditions and other pertinent information may be absent from the chart. Therefore, try to gather this and document it. Caveat: some of their medical sub-specialists may have already screened them (via telephone or video visit) for COVID-19 and may be referring them to us for a face-to-face visit, rather than a video visit (Ex: 55 y.o. man referred to us from the pulmonary clinic with chronic bronchiectasis calling for slightly worsened shortness of breath and subjective chills (no thermometer at home)-> not ill enough to send to the ED but worrisome enough to warrant an exam). If this is the case, feel free to cancel the video visit and have them come in directly for a face-to-face visit at WCIMA fever/cough clinic. Feel free to call the specialist to clarify their referral reason if the documentation is unclear; warm handoffs are especially critical for complicated patients and when balancing safety with the need to conserve PPE
- Deciding how to manage the patient: See Video Visit # 3
- Medications: See Video Visit # 3
- Counseling for isolation with and without testing: See Video Visit # 3
- Face-to-Face Visit at WCIMA Fever/cough clinic: See Video Visit # 3; staff will tell patient where we are located.
- Documentation: – see visit # 3.
- Follow up: - see visit # 3

Video Visit Type # 6 – Post-Discharge Follow-Up Visits for COVID-19 patients from inpatient units and the ED

This video visit is for a *WCIMA or non-WCIMA patient* who needs to be seen for a post-discharge visit after a hospitalization or ED visit for COVID-19. The goal of this visit is to ensure that the patient is stable and recovering at home and does not need to be sent back to the ED.

- Unique things to keep in mind: You will find these patients scheduled on the "**COVID Post Discharge**" resource. Please choose this "provider" from the HT4 list in order to see who you are scheduled for.
- These patients will have post-discharge calls by an NP or an MA, who are calling patients up to 7 days post discharge. Their notes should be in Epic and will be helpful to

review before the visit. Also, many patients will have been sent home with pulse oximeters and sometimes oxygen.

- History taking: Read the discharge summary from the inpatient unit or the ED and the follow-up call notes. Then take a focused history during the visit that includes a verification of their hospital or ED course, medications they are currently taking, and their symptoms post-discharge. Try to get a sense of their clinical trajectory since discharge (improving, same, worse). If they have an O2 tank, ask how many liters they have been using at rest, when walking around the home, and at night.
- Vitals and physical exam: Ask them to report on their current O2 saturation. Watch their respirations while they speak with you. Assessments for dyspnea (at rest or with minimal exertion) and for exertional hypoxia (< 92%) should be performed. Ask about their other vital signs if they have it. Compare vitals from the video visit to their last inpatient or ED visit day and record the change.
- Documentation elements: Since we currently do not have a separate Epic smart phrase for this visit, please use the *.COVID amb assmt smart phrase and smart text* to structure your note and orders. *Also add the following elements:* a) Type of hospital encounter (inpatient vs ED); b) Admission date; c) Discharge date; d) ICU stay (yes/no); e) Intubation (yes/no); f) Medications given during hospitalization or ED stay; g) Medications and equipment sent home with; h) PMD.
- Deciding how to manage the patient: *You will need to decide whether this person falls into one of three categories*: ***(a)*** they appear stable and can stay at home and have continued follow-up video visits with WCIMA (*See below*); ***(b)*** they appear worse than they did at discharge and they need to go back to the ED; ***(c)*** they appear worse than they did at discharge and they need to see us at CCF clinic (for labs/reassessment)
- Medications: Assist with refills or new Rx's for those they were unable to fill.
- Follow up visits: For patients who are stable and/or improving after discharge, you will need to decide how frequently they require a follow-up video visit. Remember that many patients with COVID have a second decompensation around day #8-12 of symptoms so consider a second check-in point if they have not passed this timeframe. *Route a message to "p wcima blue front desk*" indicating when you can see them. If you are out or redeployed, ask that they be scheduled on the "COVID Post Discharge schedule". For patients who appear worse and you feel need to be seen at WCIMA CCF clinic, *route a message to "p wcima blue front desk*" (see visit #3).

Video Visit Type # 7 - Health Care Personnel with COVID-19 like symptoms

This video visit is to assess NYPH health care workers who have presumed COVID-19. Before conducting this video visit, please first consult the 'Guidance for NYP HCP with or suspected COVID-19' on the NYP Infonet, last updated April 10, 2020. Testing capacity has expanded to allow testing for symptomatic healthcare personnel (HCP). This change may allow those with mild/atypical symptoms (e.g. without fever or cough) to return to work sooner if they do not have COVID. HCP still have to contact Workforce Health and Safety (WHS) who will then direct employees to choose a campus where they wish to be better where they will first undergo a video visits with a provider at that campus.

- Purpose of the video visit: Your goal will be to decide if the HCP has: ***(a)*** COVID and is ill enough to send to the ED (e.g. resting dyspnea, dyspnea with minimal exertion, AMS, etc). If so, testing will be done in the ED; ***(b)*** COVID, not ill enough to refer to the ED but concerning enough to require a CCF evaluation (e.g. to do resting and exertional O2 sat, physical exam, XRAY, labs and COVID-19 testing); ***(c)*** COVID but well enough to stay at home and self-isolate (which reduces unnecessary exposure and conserves swabs/PPE ***(d)*** mild or atypical symptoms that may or may not be COVID (e.g. cough and sore

throat without fever). Ruling COVID out will help to determine when they can return to work.

- History taking and documentation: Include the date of illness onset, symptoms, exposure to individuals with known COVID, and PPE at the time of exposure.
- Deciding how to manage the patient: Referrals to CCF should follow the above process (send a message to the '*p wcima blue front desk'* pool to schedule the patient). If you decide to refer the patient to the testing tent, please use the "**WCM COVID-19 Tent Testing**" order set. This contains a referral to the staff scheduling for the tent and a *future* ordered SARS COV-2 test which staff members at the tent can release for use.
- For patients you decide do not need testing because results are unlikely to alter mgmt, advise that future availability of serologic antibody testing can confirm their infection.
- Remember that they will need to isolate for a minimum of 7 days from the onset of symptoms and be afebrile for 72 hours without the use of antipyretics and with resolving symptoms before returning to work.
- Other points to remember: HCP should inform WH&S about their COVID test result and should call WH&S when ready to return to work; if they have been out for greater than 7 days, they will need a medical letter documenting their illness and ability to return to work. HCP should wear a face masks for a minimum of 14 days from symptom onset. Although this may feel unnecessary to advise given "mask on" policy at NYP, they should practice this in their non-work environments as well. HCP should check their daily temperatures prior to reporting to work.
- Confidentiality: All of these visits will require that you 'Break the Glass' in Epic. Please chose the option 'patient care' for the reason. You may end up doing a video visit on a colleague or someone you know. If that is the case, remember that medical information should remain confidential.

Translation Services: Pacific Interpreters # is 1-800-876-3059. Our password is 821089, followed by #. If you are using an iPad to do a video visit, you may need to use your phone (and place on speaker) for translation to work.

Documentation in Epic: You must document a video visit in Epic with a regular note. If the visit is for COVID-19 suspected illness, you can use the "*.COVID"* smart phrase. (same template for in face visit and video visit, you can select which visit type within the template) Document the physical exam as possible. There are also patient education materials also within the COVID smart texts *"Instruction if not tested"* that include isolation instructions, etc.

Billing: You should bill most video visits as a '99213' or a '99214.'

Technical issues and troubleshooting: Common issues include:

- Screen goes black but states you are connected → log out of Epic and log back in.
- You cannot hear the patient → they may need to give the app permission to use the microphone.
- If there is a failed video visit (e.g. the screen goes black, call them and do the rest via telephone. Document that part of the visit was done without video). If the entire visit is done without video, code it as '00000.'
- If you are experiencing issues with video visits, you can contact the Epic help desk for providers at 212-746-GURU; when patients get stuck, the correct support number for them is shared as well at the time of failure. You can also Contact WCM ITS Service Desk at 212-746-4878 (x64878).
